# Case Report: Primary intracranial high-grade myofibroblastic sarcoma and literature review

**DOI:** 10.3389/fonc.2025.1525401

**Published:** 2025-04-02

**Authors:** Xiaofeng He, Min Lv, Jin Yuan, Jun He, Xuemei Du, Yang Yang, Hong Zhang, Feng Wen

**Affiliations:** ^1^ Department of Medical Oncology, The First People’s Hospital of Longquanyi District, Chengdu, Chengdu, Sichuan, China; ^2^ Department of Radiology, The First People’s Hospital of Longquanyi District, Chengdu, Chengdu, Sichuan, China; ^3^ Department of Pathology, The First People’s Hospital of Longquanyi District, Chengdu, Chengdu, Sichuan, China; ^4^ Department of Radiation Oncology and Department of Head & Neck Oncology, Cancer Center, West China Hospital, Sichuan University, Chengdu, Sichuan, China; ^5^ Abdominal Oncology Ward, Division of Radiation Oncology, Cancer Center, West China Hospital, Sichuan University, Chengdu, Sichuan, China

**Keywords:** primary intracranial, high-grade myofibroblastic sarcoma, resection, radiotherapy, case report

## Abstract

High-grade myofibroblastic sarcoma (HGMS) is exceedingly rare and highly aggressive, with a poor prognosis. Currently, there is no consensus on its definition. Wide resection is the standard of care for most patients, but clinical data on treatment outcomes remain limited. Here, we present the first reported case of HGMS originating intracranially. Surgical excision of the tumor was performed, followed by adjuvant radiotherapy with a total dose of 60 Gy in 30 fractions. As of November 2024, the patient had achieved 24 months of recurrence-free survival. This case may provide new evidence that could be useful for the treatment of rare primary intracranial HGMS.

## Introduction

Malignant sarcoma composed of myofibroblasts or exhibiting myofibroblastic differentiation is diagnosed as myofibroblastic sarcoma (MS) (1). It is exceedingly rare, typically low-grade, and most commonly arises in the head and neck, extremities, or trunk (1, 2). MS is graded according to the Fédération Nationale des Centres de Lutte Contre le Cancer (FNCLCC) system, with recommendations to classify it at least into low (FNCLCC 1) versus high (FNCLCC 2 and 3) grades due to implications for neoadjuvant therapy (3). However, the 2020 World Health Organization (WHO) classification of soft tissue tumors continues to list only low-grade MS (LGMS) and does not establish a consensus on the definition of high-grade MS (HGMS), in contrast to the 2013 WHO classification (4). HGMS is primarily diagnosed through cytomorphological analysis combined with immunophenotyping. It is more aggressive and has a higher rate of recurrence and metastasis compared to LGMS (5). Surgical resection of the tumor and adjacent structures is the standard of care for most patients; however, clinical data on the outcomes of other treatments, such as chemotherapy, radiotherapy (RT), and targeted therapy (6), remain limited. Previous studies on HGMS are rare. Herein, we describe a patient with HGMS (FNCLCC 2) originating intracranially. To the best of our knowledge, this is the first reported case of primary intracranial HGMS.

## Case report

**Figure 1:**
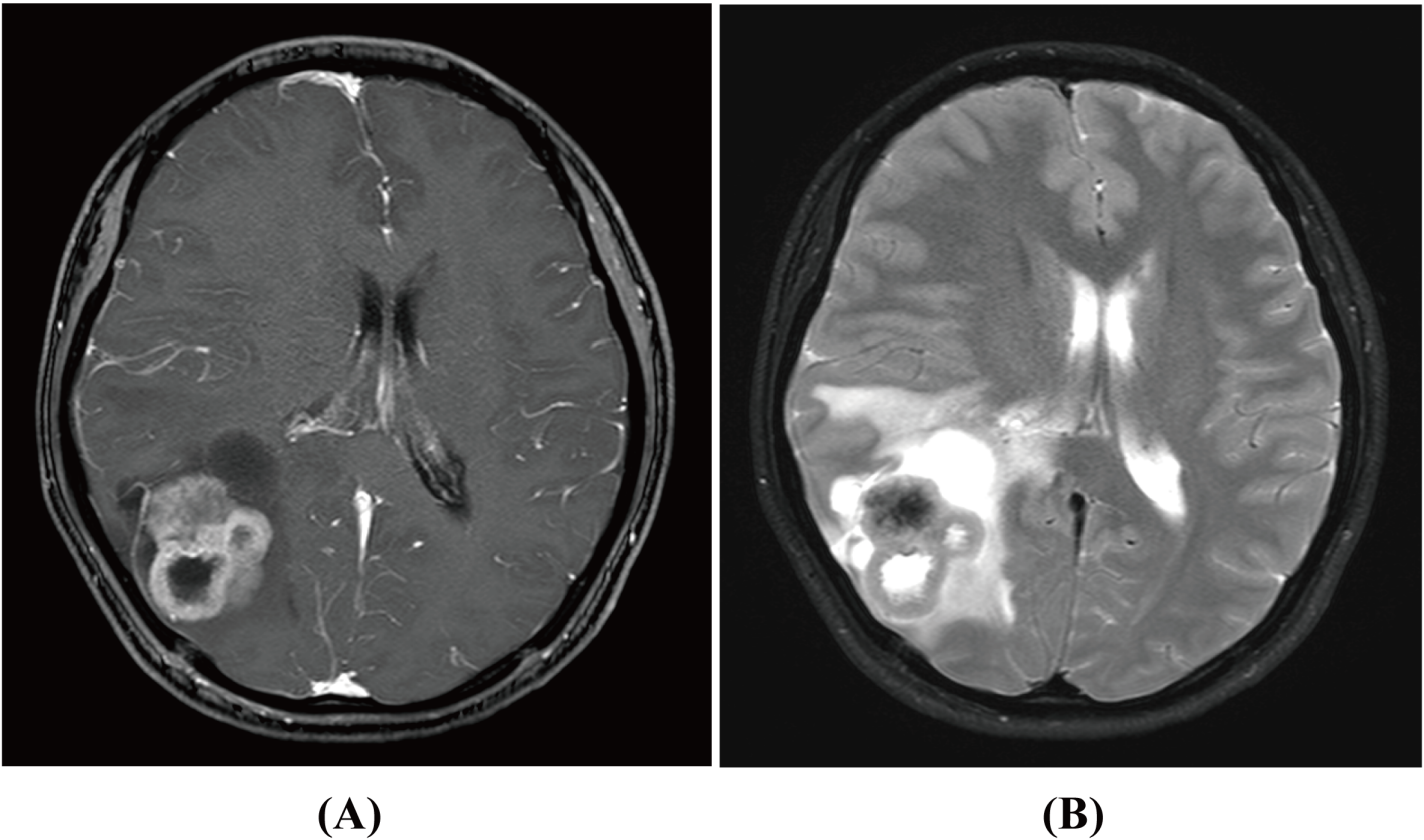
Magnetic resonance imaging (MRI) scan of primary intracranial myofibroblastic sarcoma in contrast-enhanced T1WI **(A)** and T2WI **(B)** before surgery.

In October 2022, a 23-year-old woman was admitted to our hospital with a 2-month history of progressive headaches and a 2-week history of diplopia. Physical examination revealed binocular diplopia but no other neurological abnormalities. Laboratory tests and systemic evaluations were unremarkable. Magnetic resonance imaging (MRI) scan of the head showed a 5.2 cm × 4.0 cm × 6.1 cm cystic mass with irregular margins located in the right fronto-parieto-occipital lobe (**Figures 1A, B**). The mass appeared isointense with low mixed signals on T1-weighted imaging (WI), isointense to hyperintense with low mixed signals on T2-weighted imaging (T2WI), and hyperintense on fluid-attenuated inversion recovery (FLAIR). Contrast-enhanced T1WI revealed asymmetric ring-like enhancement. Moreover, the right lateral ventricle was compressed, and the midline was slightly shifted to the left. As the lesion originated intracranially, only resection of the space-occupying mass in the right fronto-parieto-occipital lobe was performed on 27 October 2022. Upon cortical incision, a well-circumscribed, firm, vascularized parenchymatous tumor was exposed. The tumor had a gray outer surface and a red inner surface. It was large, measuring approximately 5 cm × 6 × 7 cm, and deeply located within the brain, with a distinct cyst wall in its anterior portion.

**Figure 2:**
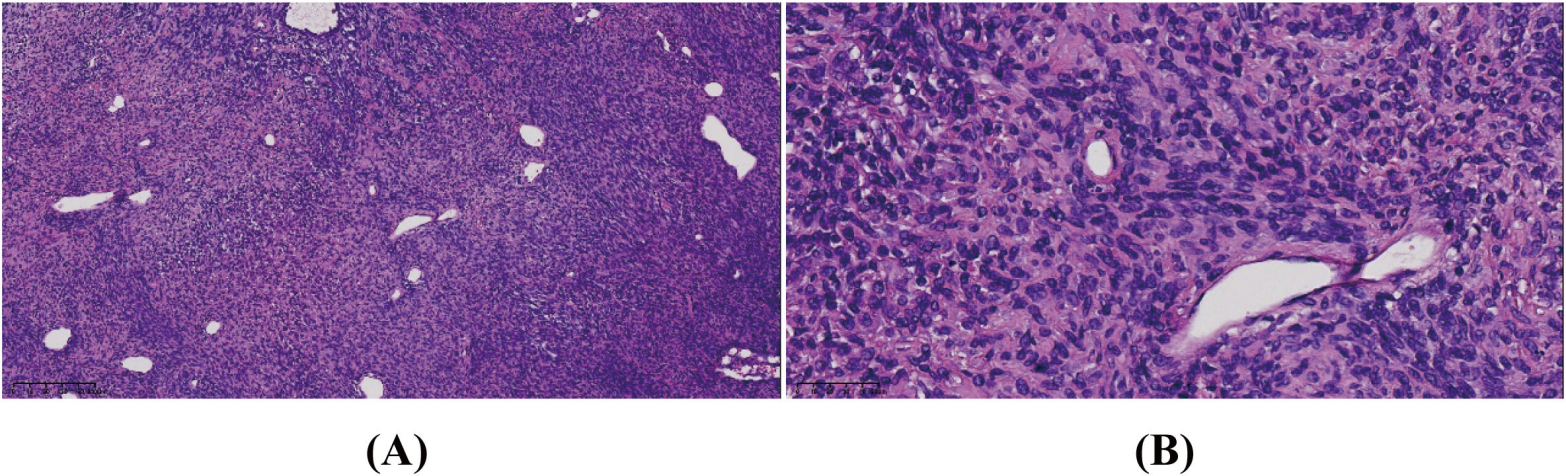
The tumor consisted of short spindle cells with hyperchromatic, distorted nuclei, an increased nuclear-to-cytoplasmic ratio, and marked nuclear atypia. Some nucleoli were seen. Hematoxylin–eosin stain. Original magnification: **(A)** × 10 and **(B)** × 40.

Intraoperative frozen section analysis revealed a tumor composed of short spindle cells with hyperchromatic, distorted nuclei, an increased nuclear-to-cytoplasmic ratio, and marked nuclear atypia. Some nucleoli were also observed (**Figures 2A, B**). Postoperative immunohistochemical staining showed positivity for desmin, focal positivity for CD34 and somatostatin receptor 2 (SSTR2), and negativity for epithelial membrane antigen (EMA), S-100, CD99, signal transducer and activator of transcription 6 (STAT6), and smooth muscle actin (SMA). Additionally, the Ki-67 index ranged from 3% to 20%. The cytomorphology and immunophenotyping suggested a rare mesenchymal tumor. Further immunohistochemical analysis revealed that the tumor cells were positive for tri-methylation of histone H3 at lysine 27 (H3K27me3), partially positive for caldesmon, and negative for glial fibrillary acidic protein (GFAP), oligodendrocyte lineage transcription factor 2 (Olig-2), myogenin, myogenic differentiation 1 (MyoD1), and SRY-related HMG-box 10 (SOX10). Genetic testing revealed no mutation in the Dicer 1, Ribonuclease III (*DICER*) gene, no translocation of the Ewing Sarcoma breakpoint region 1/EWS RNA binding protein 1 (*EWSR1*) gene, and no amplification of the Murine Double Minute 2 (*MDM2*) gene in the tumor cells. Based on these findings, the tumor was ultimately diagnosed as MS and classified as grade 2 according to the FNCLCC system. Since no primary lesions were present in other organs, a diagnosis of primary intracranial HGMS (FNCLCC 2) was established.

**Figure 3:**
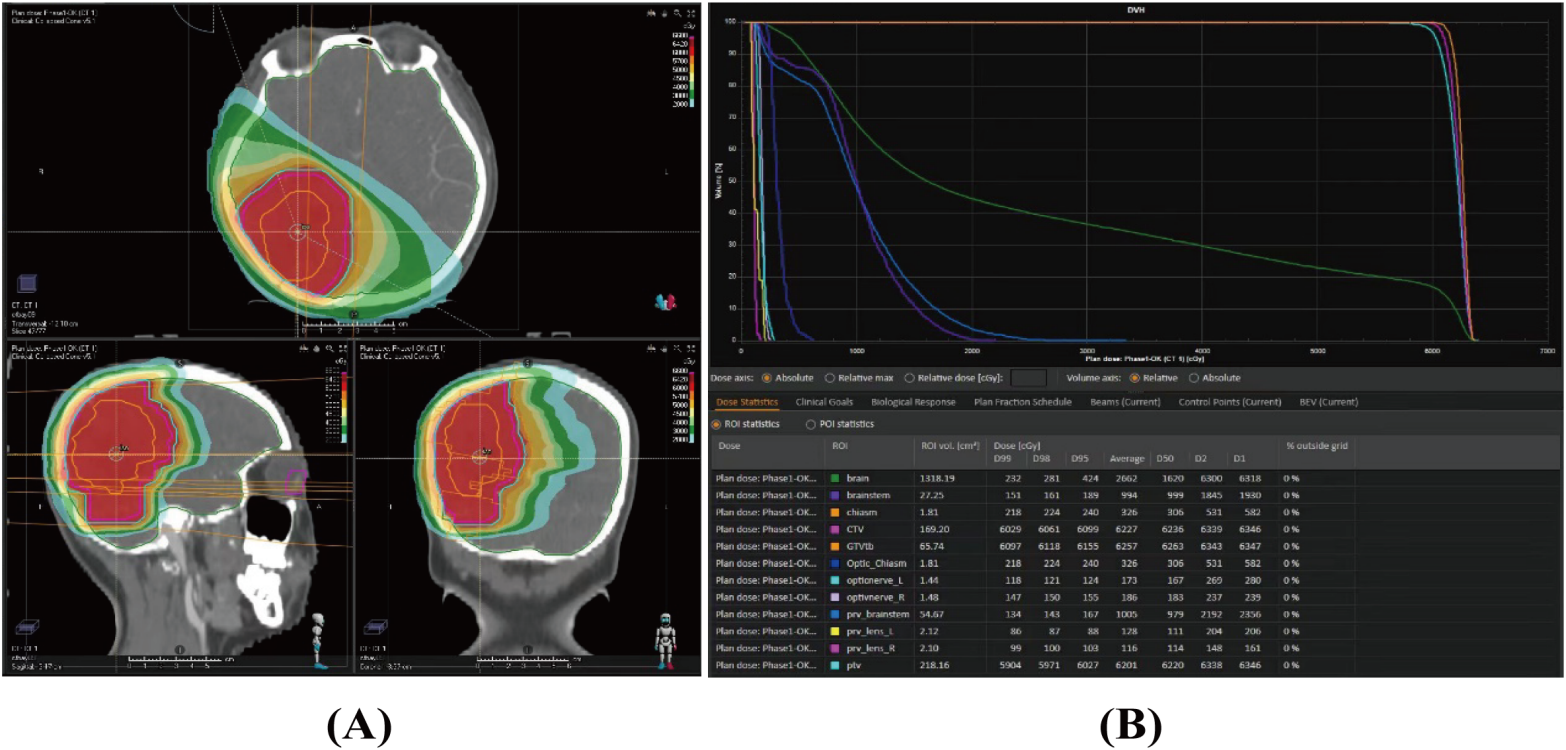
Dose distribution **(A)** and dose–volume histogram **(B)** of the radiotherapy plan for the case.

Given the rarity and aggressiveness of HGMS, the tumor’s relatively large size, and the patient’s young age, a sarcoma multidisciplinary team (MDT) discussion was conducted postoperatively. After thorough deliberation, Local RT was chosen to reduce the risk of local recurrence while minimizing systemic side effects. Chemotherapy and targeted therapy were not recommended due to their limited efficacy across the blood–brain barrier and the absence of detectable genetic mutations. Additionally, a comprehensive discussion with the patient was held before finalizing the treatment plan, considering the potential risk of cognitive dysfunction associated with local radiotherapy. The patient subsequently received adjuvant intensity-modulated radiotherapy (IMRT). The clinical target volume (CTV) encompassed the entire tumor bed, as defined by the operative record and postoperative MRI, with an additional 1.5-mm margin. The planning target volume (PTV) included the CTV plus a 5-mm margin (**Figure 3A**). A total dose of 60 Gy was delivered to the PTV in 30 fractions (**Figure 3B**) over 6 weeks, from 15 February to 28 March 2023.

Since no specific serum tumor marker exists for HGMS, imaging served as the primary follow-up strategy in this case. The protocol included contrast-enhanced head MRI, along with contrast-enhanced thoracic and abdominal CT. These imaging studies were conducted 1 month after radiotherapy, then every 3 months during the first 2 years, biannually until 5 years posttreatment, and annually thereafter. Additionally, whole-body bone scans and PET/CT scans were recommended when clinically indicated. The tumor responded well to treatment, with no signs of relapse or metastasis observed during the 24-month postoperative follow-up period.

## Discussion

MS is an exceptionally rare malignancy, with current understanding predominantly derived from case reports and small series. First described in 1978 as a low-grade entity (LGMS) by Vasudev et al. (7), this tumor type was later refined by Mentzel et al. (8) and has undergone incremental nosological clarification in recent decades. Notably, the 2020 WHO classification retains LGMS as the only recognized myofibroblastic sarcoma subtype (4, 9), highlighting the persistent diagnostic ambiguity surrounding HGMS.

In this study, we identified literature reports published between 2000 and 2024 using the search terms in PubMed. We searched for synonyms of “high-grade myofibroblastic sarcoma” and “myofibroblastic sarcoma” and reviewed reference lists of all included studies for additional sources. Our case was also incorporated into this review. Ultimately, only nine cases were analyzed, primarily from case reports (**Table 1**) (6, 10–16). We evaluated age, gender, tumor location, tumor size, tumor grade, treatment method, chemotherapy, local recurrence, and outcomes.

**Table 1:** Summary of the clinical features of eight previously reported HGMS cases and the present case.

No.	Authors	Number of cases	Age (years)	Sex	Location	Size (cm)	Grade	Treatment	Chemotherapy	Total follow-up (months)	LR	Oncological outcome	Year of publication
1	Joerger et al. (10)	1	78	M	Prostate	N/A	Grade 3	CT	Liposomal doxorubicin	17	No	AWD	2002
2	Koga et al. (11)	1	62	M	Pericardium	8.1	High grade	E+CRT	Doxorubicin and ifosfamide	6	No	AWD	2008
3	Anastasiou et al. (12)	1	86	M	Paratesticular soft tissue	N/A	Grade 2	E		6	No	NED	2014
4	Wen (13).	1	25	M	Liver	18	FNCLCC grade 3	WE		6	No	NED	2017
5	Zhao et al. (6)	1	57	F	Pleura	10.9	Grade 3	CRT	Epirubicin and ifosfamide	6	No	AWD	2020
6	Sharma et al. (14)	1	46	F	thoracic spine	N/A	High grade	CRT	Doxorubicin and cisplatin	9	Yes	AWD	2020
7	Velez Torres et al. (15)	1	79	M	Left vocal cord	1.2	Grade 3	E+RT		24	No	NED	2021
8	Harada et al. (16)	1	66	M	Unknown	20	FNCLCC grade 3	CT	Doxorubicin	3	Yes	DOD	2023
9	Present case	1	23	F	Brain parenchyma	6.1	FNCLCC grade 2	E+RT		24	No	NED	present

*HGMS*, high-grade myofibroblastic sarcoma; M, male; F, female; N/A, not applicable; E, excision; WE, wide excision; CT, chemotherapy; RT, radiotherapy; CRT, chemoradiotherapy; LR, local recurrence; NED, no evidence of disease; AWD, alive with disease; DOD, death of disease.

Our systematic review revealed a male predominance (66.7%) with a median age at diagnosis of 62 years. Unlike the historical LGMS predilection for head/neck regions (1, 2), 66.7% (six out of nine) of HGMS cases arose in the trunk, while only 22.2% (two out of nine) involved head/neck. This anatomical divergence from LGMS patterns may reflect distinct biological behavior or ascertainment bias. Tumor size averaged 10.7 cm (range: 1.2–20 cm), aligning with aggressive sarcomas. The etiological analysis identified only one radiation-associated case (postprostate radiotherapy) (10), underscoring the need for larger cohorts to better delineate risk factors.

Immunohistochemical profiling revealed variable expression of desmin (11–13, 15), SMA (6, 11–13, 15, 16), and vimentin (13, 16), with no pathognomonic markers identified. The molecular mechanisms underlying HGMS remain incompletely understood due to its rarity; however, emerging evidence implicates several key pathways: (1) Anaplastic lymphoma kinase (ALK) dynamics—while ALK fusions characterize inflammatory myofibroblastic tumors (IMT) (17), HGMS typically lacks rearrangements. However, rare cases of ALK overexpression suggest a potential progression from precursors with ALK alterations. (2) TP53 inactivation: studies have shown that TP53 mutations or deletions are common in undifferentiated pleomorphic sarcoma (UPS) (18, 19), leiomyosarcoma (LMS) (19, 20), and osteosarcoma (21). These genetic alterations correlate with both shorter disease-free survival and increased response to anthracyclines in sarcomas (19). (3) MDM2 amplification: although this TP53 antagonist is prevalent in liposarcoma and osteosarcoma (22), no detectable amplification was found in our cohort; however, systematic screening remains warranted. (4) Phosphatidylinositol 3-kinase/protein kinase B/mammalian target of rapamycin (PI3K/Akt/mTOR) activation: while direct mechanistic evidence is lacking, dysregulation of this pathway in sarcomas (23, 24) may contribute to HGMS progression.

Notably, our case excluded DICER1 mutations (associated with pediatric central nervous system tumors) (25–27), EWSR1 rearrangements (a hallmark of Ewing sarcoma) (28), and MDM2 amplification (common in liposarcoma and osteosarcoma) (22), highlighting the molecular distinctiveness of HGMS.

In our study, complete surgical resection with negative margins (achieved in 55.6% of cases) was the cornerstone of management, with three out of five resected patients remaining disease-free at 6–24 months. Adjuvant RT and chemotherapy (doxorubicin-based regimens in 55.6%) appeared beneficial, with only one reported mortality. These findings align with sarcoma management principles, where wide excision and multimodal therapy optimize outcomes.

HGMS resides within the fibroblastic or myofibroblastic spectrum, which encompasses a group of rare tumor types with often-overlapping clinicopathologic features, ranging in biological potential from benign to overtly malignant (4, 29). Among these, IMT is a common type of intermediate (locally aggressive) tumor composed of myofibroblasts, frequently harboring ALK or ROS1 fusions (17). Local recurrence is common, and ALK inhibitors (e.g., crizotinib) have shown promise in advanced cases. Adult-type fibrosarcoma is a high-grade spindle cell sarcoma characterized by a herringbone architecture, poor prognosis, and a lack of targetable drivers (4). Myxofibrosarcoma, a malignancy with curvilinear vessels and myxoid stroma, often exhibits complex genomic alterations and a high propensity for local recurrence (30). Unlike LGMS, which lacks recurrent cytogenetic alterations (29), HGMS demonstrates TP53/MDM2/PI3K axis perturbations, suggesting a divergent pathogenesis.

Although this study represents the largest aggregation of HGMS cases, it inherits limitations inherent to retrospective case synthesis, including heterogeneous reporting, selection bias, and insufficient molecular profiling. Tumor heterogeneity and therapeutic variability hinder definitive conclusions regarding prognostic factors. Therefore, prospective registries integrating next-generation sequencing (e.g., for TP53 and PI3K pathway alterations) and standardized treatment protocols are essential. These approaches are crucial for transforming HGMS from a poorly understood condition to a malignancy with evidence-based management strategies.

In conclusion, our analysis advances the characterization of HGMS as a trunk-predominant sarcoma affecting older men, necessitating aggressive resection and multimodal therapy. The molecular overlaps with TP53-driven sarcomas and PI3K pathway activation provide a rationale for targeted therapy trials. The presented intracranial HGMS case—potentially the first report—further illustrates the tumor’s anatomical versatility. Only through international collaboration and molecular profiling can this enigmatic entity transition from histopathological curiosity to a biologically defined therapeutic target.

## Data Availability

The datasets presented in this study can be found in online repositories. The names of the repository/repositories and accession number(s) can be found in the article/supplementary material.
